# A New Family of Bacteriolytic Proteins in *Dictyostelium discoideum*

**DOI:** 10.3389/fcimb.2020.617310

**Published:** 2021-02-03

**Authors:** Cyril Guilhen, Wanessa C. Lima, Estelle Ifrid, Xenia Crespo-Yañez, Otmane Lamrabet, Pierre Cosson

**Affiliations:** Department of Cell Physiology and Metabolism, Faculty of Medicine, Centre Médical Universitaire, University of Geneva, Geneva, Switzerland

**Keywords:** *Dictyostelium discoideum*, protein purification, bacteriolytic proteins, *Klebsiella pneumoniae*, intracellular killing, Bacteriolytic *D. discoideum* A (BadA)

## Abstract

Phagocytic cells ingest and destroy bacteria efficiently and in doing so ensure the defense of the human body against infections. Phagocytic *Dictyostelium discoideum* amoebae represent a powerful model system to study the intracellular mechanisms ensuring destruction of ingested bacteria in phagosomes. Here, we discovered the presence of a bacteriolytic activity against *Klebsiella pneumoniae* in cellular extracts from *D. discoideum.* The bacteriolytic activity was detected only at a very acidic pH mimicking the conditions found in *D. discoideum* phagosomes. It was also strongly decreased in extracts of *kil1* KO cells that were previously described to kill inefficiently internalized bacteria, suggesting that the activity observed *in vitro* is involved in killing of bacteria in phagosomes. We purified a fraction enriched in bacteriolytic activity where only 16 proteins were detected and focused on four proteins selectively enriched in this fraction. Three of them belong to a poorly characterized family of *D. discoideum* proteins exhibiting a DUF3430 domain of unknown function and were named BadA (Bacteriolytic *D. discoideum* A), BadB, and BadC. We overexpressed the BadA protein in cells, and the bacteriolytic activity increased concomitantly in cell extracts. Conversely, depletion of BadA from cell extracts decreased significantly their bacteriolytic activity. Finally, in cells overexpressing BadA, bacterial killing was faster than in parental cells. Together these results identify BadA as a *D. discoideum* protein required for cellular bactericidal activity. They also define a new strategy to identify and characterize bactericidal proteins in *D. discoideum* cells.

## Introduction

Phagocytic cells represent the first line of defense of the human body against invading microorganisms. They ingest and kill microorganisms, and protect the body from harmful infections. The cellular mechanisms that allow cells to kill ingested bacteria have been intensely studied since the first phagocytes were described by Metchnikov in 1882 (Yv and Dv, 2016). The first aim of this collective enterprise is to unravel how phagocytes kill bacteria, in order to better understand how some pathogens escape destruction by phagocytic cells and mount harmful infections. The second aim is to identify new mechanisms that would allow to destroy selectively bacteria in the human body and could represent tools of biomedical value in the prevention or treatment of bacterial infections.

Phagocytic cells exist in many animals, where they ensure innate defense against infecting microorganisms. They are also found as unicellular amoebae in the environment, for which phagocytosis is a means to feed upon ingested microorganisms. Amoebae and mammalian phagocytes apparently use largely similar molecular mechanisms to bind, ingest, and kill microorganisms (Cosson and Soldati, 2008). In both types of phagocytes, the putative effectors killing microorganisms are free radicals, ions, digestive enzymes, and permeabilizing peptides (Dunn et al., 2018). However, the detailed list of effectors differs significantly in amoebae and human phagocytes. For example, phagosomes acidify rapidly to approximately pH5 in mammalian cells, but *Dictyostelium discoideum* phagosomes reach pH values below 3 (Bodinier et al., 2020). Both amoebae and mammalian phagocytes express lysozymes capable of degrading bacterial proteoglycans, but the amoeba repertoire of lysozymes is much wider and more diverse: 22 genes in *D. discoideum* genome belonging to four distinct families (Lamrabet et al., 2020) vs 11 genes in human belonging to two families (Callewaert and Michiels, 2010). Similarly, amoebae exhibit whole families of permeabilizing proteins not found in mammals (Andrä et al., 2003).

In the study of antibacterial cellular mechanisms, amoebae are used as a model phagocytic cell amenable to genetic analysis (Cosson and Soldati, 2008), but also as a source of new antibacterial proteins distinct from those found in animals. Antibacterial proteins may represent usable tools in humanity’s constant fight against infectious bacteria. Of all amoebae, the most studied so far has been the *D. discoideum* social amoeba. Its genome is sequenced and annotated and many genetic tools have been developed over decades. It has been used as a model phagocytic cell to study many unicellular traits (e.g. motility, chemotaxis or phagocytosis) as well as its starvation-induced multicellular development (Bozzaro, 2013). It has been also a powerful model system to study cellular mechanisms allowing the destruction of ingested bacteria in phagosomes. Screening of library of random mutants notably allowed the identification and characterization of Kil1 and Kil2, two gene products critically involved in the killing of ingested *K. pneumoniae* bacteria in *D. discoideum* phagosomes. Kil1 is a sulfotransferase responsible for the sulfation of sugars, a modification common to a variety of (potentially microbicidal) lysosomal proteins (Benghezal et al., 2006). Kil2 is a putative magnesium pump found in the phagosomal membrane and its activity is notably critical for optimal activity of phagosomal proteases (Lelong et al., 2011). Genetic inactivation of *kil1* or *kil2* genes lead to a strong defect in intracellular killing of *K. pneumoniae*. The effectors directly responsible for killing or lysis of bacteria in phagosomes have however not been identified so far. One of the best attempts at identifying *D. discoideum* bacteriolytic effectors has been the purification of the main *D. discoideum* lysozyme protein, named AlyA (Müller et al., 2005). The exact role of AlyA in bacterial killing, if any, has not been clearly established to date. Of note, one would expect putative *D. discoideum* phagosomal bactericidal mechanisms to function at a very low pH, since *D. discoideum* phagosomes are unusually acidic. A variety of probes have indeed been used to measure the pH of endosomal compartments (Marchetti et al., 2009), and all indicate that endocytic compartments have a pH lower than 3.5. Similarly, the pH of phagosomes has been proposed to be as low as 2.5 (Bodinier et al., 2020).

In an attempt to identify new microbicidal effectors in *D. discoideum*, we engaged in a search for bacteriolytic proteins in *D. discoideum* cell extracts. We identified the BadA (Bacteriolytic *D. discoideum* A) protein as the founding member of a new family of bacteriolytic proteins found mostly in dictyostelids and related organisms.

## Materials and Methods

### Strain and Cell Culture

*D. discoideum* cells used in this study were all derived from the DH1-10 parental strain (Cornillon et al., 2000), referred to in this study as WT. WT cells, *kil1* (Benghezal et al., 2006), *kil2* (Lelong et al., 2011), *kil1-kil2*, *alyL*, *bpiC*, *aoaH*, and *aplA* (not published) KO strains were grown at 21°C in HL5 medium (Froquet et al., 2008). Uncapsulated *K. pneumoniae* Ge (Lima et al., 2018) and capsulated *K. pneumoniae* LM21 (Balestrino et al., 2008) were grown overnight at 37°C in Luria-Bertani. A *waaQ* mutant of *K. pneumoniae* KpGe was previously described (Benghezal et al., 2006).

### Overexpression of BadA Protein

A DNA sequence was synthesized (GeneArt, Invitrogen) corresponding to the cDNA sequence of BadA (Uniprot Q86LA4) with the sequence coding the ALFA tag (SRLEEELRRRLTE) (Götzke et al., 2019) inserted at the C-terminus of BadA. The synthesized sequence was cloned in the prepSC3 expression vector (Froquet et al., 2012). The recombinant vector was transfected into WT *D. discoideum* cells and individual clones were selected in a medium containing geneticin (10 µg/mL). Individual clones were screened by western blot analysis to check for expression of the BadA-ALFA protein.

### Bacterial Lysis Assay

To measure bacterial lysis, 2×10^8^ *D. discoideum* cells were washed twice in phosphate buffer (PB: 2 mM of Na2HPO4 and 14.7 mM of KH2PO4, pH6.0) and lysed in 800 μl of lysis buffer [50 mM of sodium phosphate buffer (NaP buffer), pH3, 0.5% Triton X‐100] containing when specified protease inhibitors [20 μg/ml of leupeptin, 10 μg/ml of aprotinin, 18 μg/ml of phenylmethylsulfonyl fluoride (PMSF), and 1.8 mg/ml of iodoacetamide (IAA)]. Commercial protease inhibitor tablets were also used when indicated (Pierce #A32963). The suspension was centrifuged (30,000 g for 10 min at 4°C), and the supernatant was collected and diluted in lysis buffer with the indicated pH (from 1 to 7). Bacteriolytic activity was assessed by mixing in a microtiter plate 100 μl of cell extract (or lysis buffer as negative control) with 100 μl of overnight bacterial culture washed once in NaP buffer (to the corresponding pH) and resuspended to a final optical density at 450 nm of 0.5. The decrease in turbidity (optical density at 450 nm) over 2 h of incubation at 21°C was measured with a spectrophotometer plate reader and used to determine the bacterial lysis activity of either total cell extracts or fractions of it.

### Western Blot Analysis

10^6^ *D. discoideum* cells were pelleted and resuspended in 40 µl of reducing sample buffer [20.6% (w/v) sucrose, 100 mM Tris pH6.8, 10 mM EDTA, 0.1% (w/v) bromophenol blue, 4% (w/v) SDS, 6% (v/v) β-mercaptoethanol]. Twenty µl of each sample was migrated (200 V, 30 min) in a 4–20% acrylamide gel (SurePAGE Bis-Tris, Genscript #M00655), and transferred to a nitrocellulose membrane using a dry transfer system for 7 min (iBlot gel transfer device, Invitrogen #IB1001EU). The membranes were blocked during 2 h in PBS containing 0.1% (v/v) Tween 20 and 7% (w/v) milk, and washed three times for 5 min in PBS + 0.1% (v/v) Tween 20. The AL626 antibody specific for the ALFA epitope was produced as a mini-antibody (VHH-mouseFc) by the Geneva Antibody Facility (https://www.unige.ch/medecine/antibodies/). The membranes were incubated with the primary antibody AL626 (dilution 1:50) (Guilhen, 2020; Lima and Cosson, 2020) for 1 h at room temperature, then washed three times for 5 min. The membranes were then incubated with horseradish peroxidase-coupled goat anti-mouse IgG (Biorad #170-6516, dilution 1:3,000) or anti-rabbit IgG (Sigma-Aldrich #A8275, dilution 1:3,000 and washed twice for 5 min and once for 15 min in PBS-Tween. The signal was revealed by enhanced chemiluminescence (ECL) (Millipore) using a PXi-4 gel imaging systems (Syngene).

### Purification Procedures

10^9^ *D. discoideum* cells were pelleted, washed in phosphate buffer, and lysed in lysis buffer at pH3 to a final volume of 1.7 ml. Nuclei and debris were pelleted (30,000 g for 10 min at 4°C) and the supernatant was recovered and mixed with 200 µl of anion exchange resin (Q Sepharose Fast Flow; Sigma #17-0510-10) pre-washed in lysis buffer at pH3. After 1 h of incubation at 4°C on a wheel, the anionic resin was recovered and washed five times with 1 ml of lysis buffer at pH3. Negatively charged proteins attached to the resin were then eluted with 500 µl of NaP buffer at pH3 with increasing concentration of NaCl from 0.05 to 0.5 M of NaCl with an increment of 50 mM. Fractions presenting the highest bacteriolytic activity (e.g., fractions 0.15, 0.20, 0.25, and 0.30 M of NaCl) were mixed, buffered at pH7 and loaded on a size-exclusion chromatographic (SEC) column (Superdex 200 10/300 GL; Sigma #17-5175-01). The column was equilibrated with NaP buffer at pH7 and eluted with the same buffer at a rate of 18 ml/h and 0.5 ml by fraction.

### Protein Silver Nitrate Staining

To visualize the protein profiles of total cell extracts or fractions of it, proteins were separated according to their size in a 4–20% acrylamide gel (SurePAGE Bis-Tris, Genscript #M00655) and then silver-stained. The gel was fixed with a fixative solution (40% (v/v) ethanol, 10% (v/v) acetic acid) for 30 min and incubated overnight with a solution containing 30% (v/v) of ethanol, 0.26% (v/v) of glutaraldehyde, 6.8% (w/v) of sodium acetate trihydrate and 0.2% (w/v) of sodium thiosulfate pentahydrate. After three washes of 5 min in deionized water, the gel was incubated for 40 min with a solution containing 0.1% (w/v) of silver nitrate and 0.1% (v/v) of formaldehyde. After a brief wash of 10 s in deionized water, the gel was incubated 1–5 min with a developmental solution containing 2.5% (w/v) of sodium carbonate and 0.01% (v/v) of formaldehyde until the appearance of proteins. The developmental reaction was stopped by the incubation of the gel with a solution containing 1.46% (w/v) of EDTA dihydrate.

### Immunoprecipitation

10^8^ *D. discoideum* cells were pelleted, washed in phosphate buffer and lysed in PBS + 0.5% (v/v) Triton X‐100 containing protease inhibitors [20 μg/ml of leupeptin, 10 μg/ml of aprotinin, 18 μg/ml of phenylmethylsulfonyl fluoride (PMSF) and 1.8 mg/ml of iodoacetamide (IAA)] to a final volume of 1 ml. Nucleus were pelleted and 800 μl of the recovered supernatant was mixed with 50 µl of uncoupled protein A pre-washed in PBS + 0.1% (v/v) Tween 20. After 1 h of incubation at 4°C on a wheel, the cleared lysate was recovered and mixed with 50 µl of ALFA selector PE resin (Nanotag Biotechnologies #N1510) pre-washed in PBS + 0.1% (v/v) Tween 20. After 1 h of incubation at 4°C on a wheel, the resin was recovered and washed 5 times with 1 ml of PBS + 0.1% (v/v) Tween 20. Proteins attached to the resin were finally eluted with 100 µl of PBS containing 200 µM of ALFA peptide (Nanotag Biotechnologies #N1520-L) (Götzke et al., 2019).

### Protein Identification by ESI-LC-MSMS

#### Sample Preparation

Protein samples were concentrated and purified by 1D gel electrophoresis (SDS- PAGE; stacking gel method) using a home-made 4% bis-acrylamide stacking gel and a 12% bis- acrylamide resolving gel. Briefly, samples were loaded and migration was performed at 70 V until the proteins enter the resolving gel from around 5mm. Protein gel was then stained with coomassie blue G-250. For each sample, the 5 mm-band containing all proteins was cut and in-gel digested as follows. Gel pieces were destained by incubation in 200 μl of 50% acetonitrile (AcN) in 50 mM ammonium bicarbonate (AB) for 15 min at room temperature. Proteins were reduced by incubation of gel pieces for 30 min. at 50°C in 100 μl of 10 mM DTT in 50 mM AB. DTT solution was then replaced by 100 μl of 55 mM iodoacetamide in 50 mM AB and protein were alkylated by incubation of the gel pieces for 30 min at 37°C in the dark. Gel pieces were then washed for 15 min with 100 μl of 50mM AB and for 15 min with 100 μl of 100% AcN. Gel pieces were then air dried for 15 min at room temperature. Dried pieces of gel were rehydrated for 45 min at 4°C in 40 μl of a solution of 50 mM AB containing trypsin at 10 ng/μl and 0.01% of Protease Max (PM) Surfactant trypsin enhancer (Promega). Subsequently, 10 μl of 0.01% of PM was added before incubating the samples for 1 h at 50°C. Supernatant was transferred to a new polypropylene tube and an additional peptide extraction was performed with 50 μl of 20% TFA for 15 min at room temperature with occasional shaking. Extractions were pooled, completely dried under speed vacuum and stored at -20°C.

#### ESI-LC-MSMS

Samples were diluted in 15 μl of loading buffer (5% CH3CN, 0.1% FA) and 4 μl were injected on column. LC-ESI-MS/MS was performed on a Q-Exactive Plus Hybrid Quadrupole-Orbitrap Mass Spectrometer (Thermo Fisher Scientific) equipped with an Easy nLC 1000 liquid chromatography system (Thermo Fisher Scientific). Peptides were trapped on an Acclaim pepmap100, C18, 3 μm, 75 μmx20 mm nano trap-column (Thermo Fisher Scientific) and separated on a 75 μmx250 mm, C18, 2 μm, 100 Å Easy-Spray column (Thermo Fisher Scientific). The analytical separation was run for 90 min using a gradient of H2O/0.1% Formic acid (solvent A) and CH3CN/0.1% Formic acid (solvent B). The gradient was run as follows: 0–5 min 95% A and 5% B, then to 65% A and 35% B for 60 min, then to 10% A and 90% B for 10 min and finally stay 15 min at 10% A and 90% B. Flow rate was of 250 nL/min for a total run time of 90 min. For MS survey scans, the resolution was set to 70,000 and the ion population was set to 3×106 with an m/z window from 400 to 2,000. For data dependent analysis, up to 15 precursor ions were isolated and fragmented by higher-energy collisional dissociation HCD at 27% NCE. For MS/MS detection, the resolution was set to 17,500, the ion population was set to 1×105 with an isolation width of 1.6 m/z units.

#### Database Search

Peak lists (MGF file format) were generated from raw data using the MS Convert conversion tool from ProteoWizard. The peaklist files were searched against the *D. discoideum* Reference Database (Uniprot, release 2018-10, 12746 entries) combined with an in-house database of common contaminant using Mascot (Matrix Science, London, UK; version 2.5.1). Trypsin was selected as the enzyme, with one potential missed cleavage. Precursor ion tolerance was set to 10 ppm and fragment ion tolerance to 0.02 Da. Carbamidomethyl of cysteine was specified as fixed modification. Deamidation of asparagine and glutamine, and oxidation of methionine were specified as variable modifications. The Mascot search was validated using Scaffold 4.8.4 (Proteome Software). Peptide identifications were accepted if they could be established at greater than 12.0% probability to achieve an FDR less than 0.1% by the Peptide Prophet algorithm (Keller et al., 2002) with Scaffold delta-mass correction. Protein identifications were accepted if they could be established at greater than 60.0% probability to achieve an FDR less than 1.0% and contained at least one identified peptide. Protein probabilities were assigned by the Protein Prophet algorithm (Nesvizhskii et al., 2003). Proteins that contained similar peptides and could not be differentiated based on MS/MS analysis alone were grouped to satisfy the principles of parsimony. Proteins were annotated with GO terms from NCBI (downloaded 01 oct. 2018) (Ashburner et al., 2000).

#### RAW Data

The mass spectrometry proteomics data have been deposited to the ProteomeXchange Consortium *via* the PRIDE (Perez-Riverol et al., 2019) partner repository with the dataset identifier PXD020632 and 10.6019/PXD020632.

### Intracellular Destruction of Bacteria Visualized by Live Microscopy

The intracellular destruction of fluorescent bacteria was measured as described previously (Bodinier et al., 2020). Briefly, fluorescent bacteria and *D. discoideum* cells (density between 2 and 5.10^6^ cells/ml) were washed in PB supplemented with 100 mM sorbitol (PB-Sorbitol). Fluorescent *K. pneumoniae* KpGe bacteria were deposited in a glass-bottom well (µ-slide 8-well, IBIDI) and allowed to sediment for 5 min before the addition of *D. discoideum* cells. To image the whole cell volume at each time point, an image (brightfield and with filter for GFP) was taken in five successive focal planes with a step size of 3 µm every 30 s for 2 h with a Nikon eclipse Ti2 widefield time-lapse microscope equipped with a DS-Qi2 camera. The NIS software was used to extract the images, and Fiji was used to compile and analyze movies. For each individual bacterium, the time 0 was the time when it entered a *D. discoideum* cell and bacterial destruction was indicated by the loss of its fluorescence. Intracellular destruction of phagocytosed bacteria was calculated using the Kaplan-Meier estimator on Prism version 7.0.a Graphpad. In order to evaluate variability between independent experiments and to determine the significance of the observed differences, we calculated for each independent experiment the area under the curve (AUC) between 0 and 75 min and determined the bacterial destruction capacity of either UT or BadA-ALFA transfected cells relative to UT cells (set to 100%) (bacterial destruction capacity: (AUC_UT_/AUC_+BadA_)x100.

## Results

### A Bacteriolytic Activity in *Dictyostelium discoideum* Extracts

**Figure 1:**
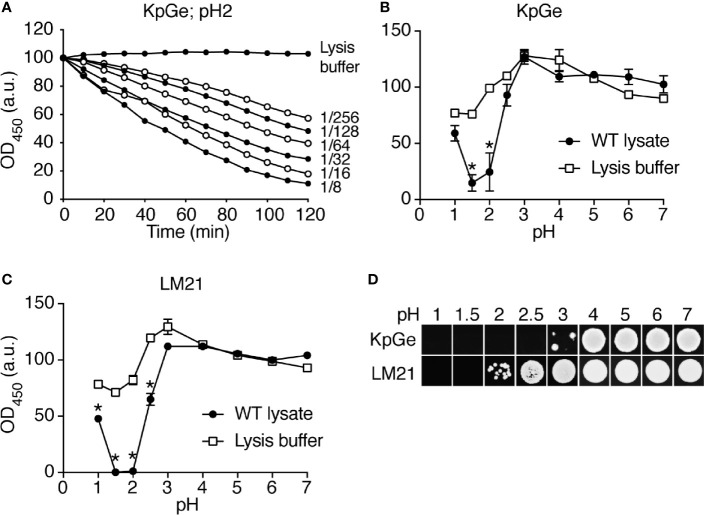
Bacteriolytic activities in *D. discoideum* cell extracts. **(A)** A cell extract from WT *D. discoideum* was serially diluted (from 1/8 to 1/256) in lysis buffer and then mixed with *K. pneumoniae* KpGe bacteria at pH2. The bacteriolytic activity was monitored over time by spectrophotometry at 450 nm (OD_450_). Results are expressed as a percentage of OD_450_ at time 0. *D. discoideum* cell extracts lysed *K. pneumoniae* KpGe bacteria at pH2. This is a set of data from a representative experiment. **(B, C)** The bacteriolytic activity was measured as described in **(A)** over a range of pH from 1 to 7. The OD_450_ values after 120 min of incubation were used to determine the effect of pH on the bacteriolytic activity of the cell extract (dilution 1/32). The bacteriolytic activity against *K. pneumoniae* KpGe *(B)* and LM21 **(C)** bacteria was maximal at pH1.5. No activity was observed above 2.5. Approximatively 25% of bacteria spontaneously lysed at pH1 and 1.5 (mean ± SEM; N = 3 independent experiments; *: p <.05; student t test). **(D)** Bacteria were washed once in phosphate buffer at the indicated pH and 10 μl were spotted on a LB agar plate to measure bacterial viability. Both *K. pneumoniae* KpGe and LM21 bacteria were dead at acidic pH (1.5 or 2) before the beginning of the bacterial lysis assay. Thus, in the conditions where the bacterial lysis is assessed, bacteria are dead.

**Figure 2:**
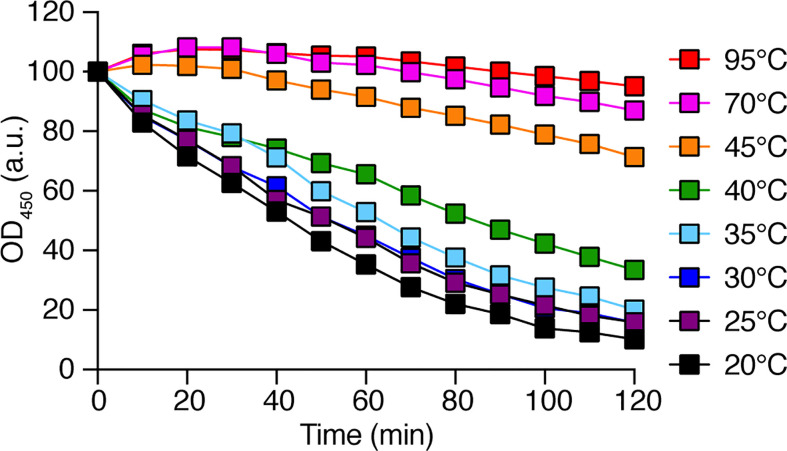
The *D. discoideum* bacteriolytic activity is sensitive to high temperature. *D. discoideum* cell extracts (dilution: 1/8) were incubated for 1 h at the indicated temperature. They were then mixed with *K. pneumoniae* KpGe bacteria at pH2 and the bacteriolytic activity was monitored overtime by spectrophotometry at 450 nm. Results are expressed as a percentage of OD_450_ at time 0. This is a set of data from a representative experiment.

In order to identify *D. discoideum* proteins involved in the intracellular destruction of ingested bacteria, we first incubated intact *K. pneumoniae* bacteria with *D. discoideum* cell extracts. We observed that the optical density of the bacterial suspension decreased as bacteria were gradually lysed (**Figure 1A**). The bacterial lysis was slower when the cell extract was diluted, and was not observed in buffer alone (**Figure 1A**). Since destruction of ingested bacteria occurs in very acidic phagosomes (pH below 3) (Bodinier et al., 2020), the initial experiments were carried out at a pH2 unless otherwise specified. When the same assay was performed at different pH values, the lytic activity was only observed at very acidic pH (below pH2.5) in agreement with the notion that this bacteriolytic activity occurs in very acidic conditions mimicking acid *D. discoideum* phagosomes (**Figure 1B**). While most experiments described in this study focused on the non-pathogenic *K. pneumoniae* KpGe strain (Lima et al., 2018), we also tested the ability of *D. discoideum* extracts to lyse the pathogenic *K. pneumoniae* LM21 strain and found that, like non-pathogenic *K. pneumoniae*, it was lysed at very acidic pH by *D. discoideum* cellular extracts (**Figure 1C**). Note that this test does not measure killing of bacteria: when bacteria were incubated in buffer at different pH and their viability assessed by plating them and allowing them to grow on LB plates, KpGe *K. pneumoniae* died at a pH lower than 4, and LM21 *K. pneumoniae* at a pH lower than 2.5–3 (**Figure 1D**). The bacteriolytic activity was not inhibited by protease inhibitors (**Supplementary Figure 1**) and was inactivated when extracts were incubated for 1h at a temperature above 40°C (**Figure 2**).

**Figure 3:**
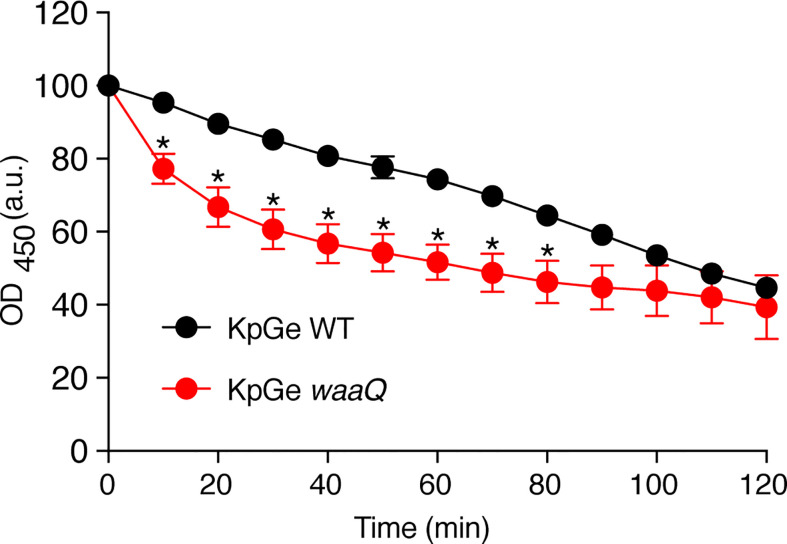
The *D. discoideum* bacteriolytic activity is more efficient against bacteria with disrupted cell walls. *D. discoideum* cell extracts (dilution: 1/32) were mixed with either WT or KO *waaQ K. pneumoniae* KpGe bacteria at pH2 and the bacteriolytic activity was monitored overtime by spectrophotometry at 450 nm. Results are expressed as a percentage of OD_450_ at time 0 (mean ± SEM; N = 4 independent experiments; *: p <.05; student t test).

If the bacteriolytic activity observed in this assay does reflect an activity in phagosomes, we would expect it to be more effective against bacteria that have been shown to be destroyed more easily in phagosomes. To test this hypothesis, we measured the lysis of a *waaQ* mutant of KpGe *K. pneumoniae*, which is killed more efficiently in *D. discoideum* phagosomes, allowing its elimination even in the phagosomes of killing-defective *kil1* KO mutant cells (Benghezal et al., 2006). Indeed, the *waaQ* mutant of KpGe was more rapidly lysed by *D. discoideum* extract (**Figure 3**). These results indicate that lysis of bacteria by *D. discoideum* extracts *in vitro* recapitulates several features of bacterial destruction in phagosomes.

### *Dictyostelium discoideum* Bacteriolytic Activity is Decreased in kil1 KO Cells

**Figure 4:**
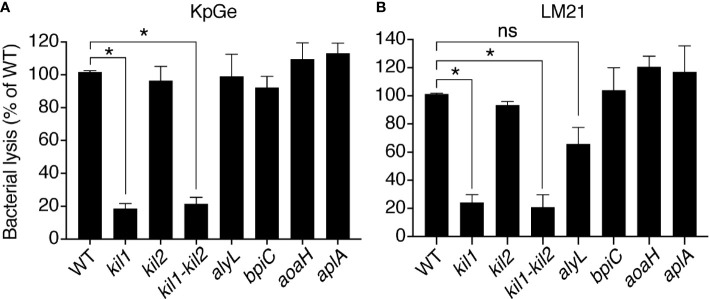
The bacteriolytic activity of *kil1* KO *D. discoideum* cells is weaker than in WT cells. Cell extracts (diluted either 8, 16, 32, 64, 128, or 256 times) from *D. discoideum* WT, *kil1*, *kil2*, *kil1-kil2*, *alyL*, *bpiC*, *aoaH*, or *aplA* KO strains were mixed with either *K. pneumoniae* KpGe **(A)** or *K. pneumoniae* LM21 **(B)** bacterial strains and bacterial lysis assessed. Results are expressed as a percentage of OD_450_ values obtained with WT amoeba after 2 h of incubation (mean ± SEM; *p < .01; student t test; for panel A: *kil1*: N = 14, *kil2*: N = 11, *kil1-kil2*: N = 12, *alyL* and *bpiC*: N = 6, *aoaH*: N = 7, *aplA*: N = 5; for panel B: N = 4).

We then measured the activity of *D. discoideum* extracts from mutant cells with a decreased ability to kill and destroy ingested *K. pneumoniae*. Genetic inactivation of *kil1* was previously shown to decreases the ability of mutant cells to kill ingested bacteria (Benghezal et al., 2006). We observed that the bacteriolytic activity was strongly decreased in extracts from *kil1* KO cells compared to extracts from WT cells (**Figure 4A**). On the contrary *kil2* KO cells kill poorly ingested bacteria because Kil2 is necessary to maintain a proper ionic composition in phagosomes, a role that would be lost in an *in vitro* bacteriolytic assay. Extracts from *kil2* KO cells exhibited a lytic activity indistinguishable from extracts of WT cells (**Figure 4A**). Together these results reinforce the notion that the bacteriolytic activity measured *in vitro* in *D. discoideum* extracts corresponds to an activity involved in bacterial killing in phagosomes.

We also tested the lytic activity from cells where known enzymes potentially lysing *K. pneumoniae* were genetically inactivated: extracts from *alyL* KO, *bpiC* KO, *aoaH* KO or *aplA* KO exhibited the same bacteriolytic activity as extracts from WT cells (**Figure 4A**), indicating that the corresponding gene products were not required for the bacteriolytic activity of *D. discoideum* cell extracts. We obtained essentially identical results when we assessed the bacteriolytic activity against the virulent LM21 strain of *K. pneumoniae* (**Figure 4B**), further suggesting that similar molecular mechanisms are responsible for the bacteriolytic activity of *D. discoideum* against different strains of *K. pneumoniae*.

### Purification of *Dictyostelium discoideum* Bacteriolytic Proteins

**Figure 5:**
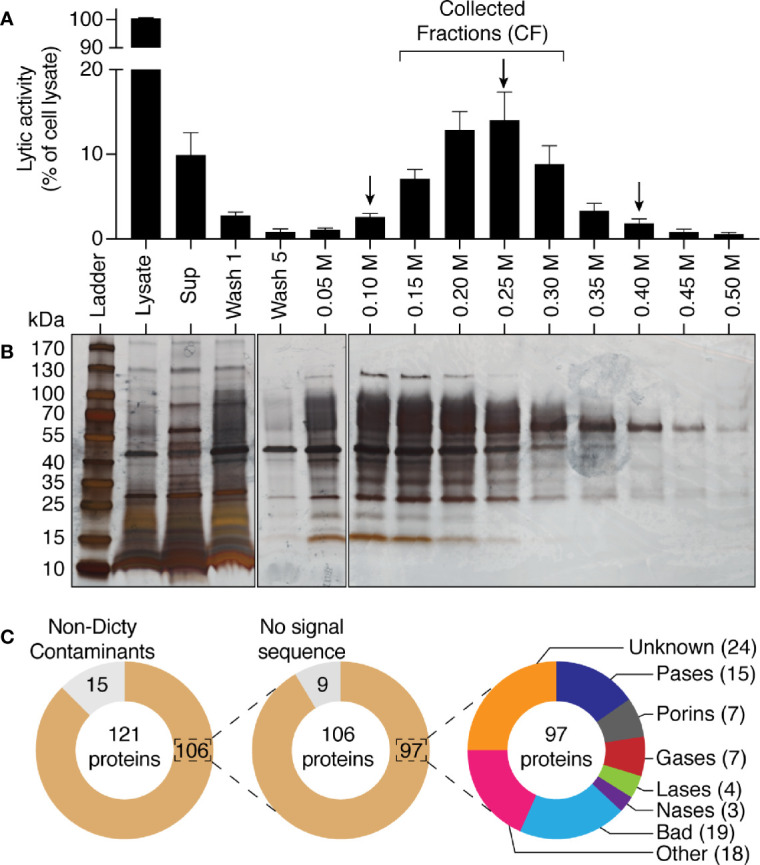
Purification by anionic exchange fractionation of bacteriolytic proteins. WT *D. discoideum* cell lysate was mixed with an anionic exchange resin at pH3. The anionic resin was washed five times and proteins fixed to the resin were eluted with a gradient of NaCl from 0.05 M to 0.5 M of NaCl. Unattached proteins were recovered in the supernatant (sup). **(A)** each fraction was tested for its bacteriolytic activity against *K. pneumoniae* KpGe bacteria. Results are expressed as a percentage of OD_450_ values obtained with total cell lysate after 2 h of incubation (mean ± SEM; N = 7 independent experiments). **(B)** Silver nitrate stained SDS PAGE gel showing protein composition pattern of each of the obtained fractions. 0.75 μl over the 1,700 μl of either the total cell lysate or supernatant were loaded in the gel. Fifteen μl over the 1,000 μl of washing steps or the 200 μl of each of the elution were loaded. Molecular mass markers are indicated at the left. **(C)** Protein composition of three fractions (indicated by a black arrow; e.g., 0.10, 0.25, and 0.40 M) were analyzed by mass spectrometry. Among the 121 proteins identified in the three samples, 15 were non- *D. discoideum* proteins (e.g., human keratin) and nine do not display a signal sequence. Of the 97 remaining *D. discoideum* proteins, 15 were proteases (Pases), 7 were porins, 7 were glycosidases (Gases), 4 were lipases (Lases), and 3 were nucleases (Nases). Finally, 43 had an unknown function (including 19 Bad proteins), and 18 had non-lytic functions.

We next attempted to purify the factor(s) responsible for the bacteriolytic activity of *D. discoideum*. Since lysosomal proteins in *D. discoideum* bear sugars that are usually sulfated or phosphorylated, we reasoned that many of them may remain negatively charged even at a pH of 3, a highly unusual property that would allow a very efficient separation from the mass of other proteins. We thus incubated *D. discoideum* extracts at pH3 with anion exchange beads, and observed that 90% of the bacteriolytic activity disappeared from the supernatant of the incubation (**Figure 5A**). Moreover, when the proteins adsorbed to the beads were eluted with increasing salt concentrations, the lytic activity was quantitatively recovered (**Figure 5A**). An aliquot of each sample was analyzed by SDS-PAGE and silver staining, revealing that this single purification step resulted in a very efficient purification of a small subset of cellular proteins (**Figure 5B**). In order to gain insight on the proteins purified in this manner, we analyzed by mass spectrometry three samples, one (250 mM NaCl) corresponding to the peak of lytic activity and two (100 and 400 mM NaCl) where lower amounts of activity were observed. A total of 121 proteins were identified in the three samples analyzed (**Figure 5C**, **Supplementary Table 1**). Fifteen proteins were not encoded by the *D. discoideum* genome (e.g., human keratin) and were likely contaminants that were not considered further. Of the remaining 106 proteins, only nine did not exhibit a signal sequence and are unlikely to be lysosomal proteins. Several of them were among the 300 most abundant *D. discoideum* proteins (actin, Gfm2, Rab7A, RapA) and presumably represent accidental contaminants. Of the 97 *D. discoideum* proteins exhibiting a signal sequence, 36 had a known lytic activity (e.g., protease or porin), 43 had an unknown function (including 19 Bad proteins, see below), and for 18, some functional information was available, but with no clear indication of a lytic function. This cursory analysis suggested that in a single step we purified a small set of proteins highly enriched in lysosomal proteins, and that contained the majority of the bacteriolytic activity of *D. discoideum* extracts.

**Figure 6:**
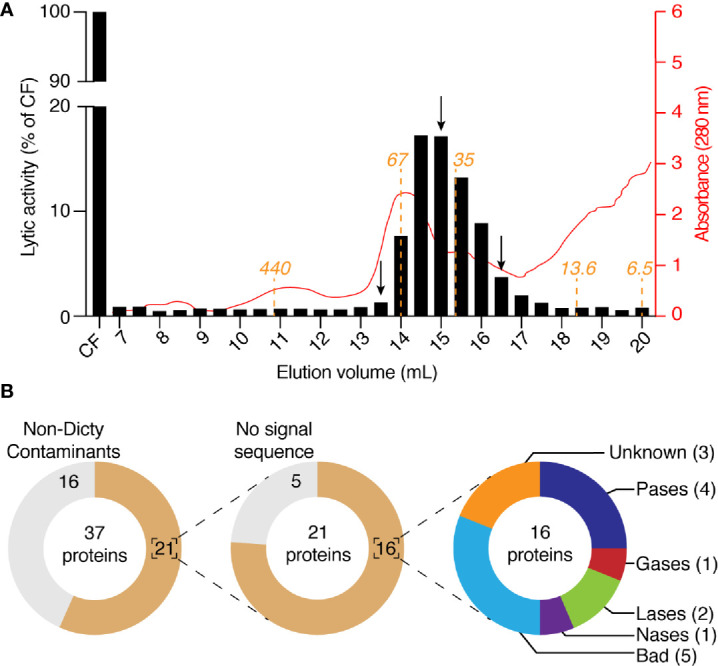
Purification by size-exclusion chromatography of bacteriolytic proteins. **(A)** The four fractions with the highest activity in **Figure 5A** (e.g., 0.15, 0.20, 0.25, and 0.30) were collected, mixed, buffered at pH7 and loaded on a size-exclusion chromatographic column. The column (Superdex 200; 10 x 300 mm) was equilibrated with NaP buffer at pH7 and eluted with the same buffer at a rate of 18 ml/h and 0.5 ml by fraction. Each of the obtained fractions was then tested for their bacteriolytic activity against *K. pneumoniae* KpGe bacteria. Results are from a single experiment, and are expressed as a percentage of OD_450_ values obtained with the four collected fractions after 2 h of incubation. Relative protein abundance in each of the obtained fractions was monitored by spectrophotometry at 280 nm. The predicted molecular weight of proteins eluted from the Superdex 200 column were indicated in orange. **(B)** Protein composition of three fractions (indicated by a black arrow; e.g., 13.5, 15, and 16.5 ml) were analyzed by mass spectrometry. Among the 37 proteins identified in the three samples, 16 were non- *D. discoideum* proteins and 5 do not display a signal sequence. Of the 16 remaining *D. discoideum* proteins, 4 were proteases (Pases), 1 was glycosidase (Gases), 2 were lipases (Lases), and 1 was nuclease (Nases). Finally, eight had an unknown function (including five Bad proteins).

**Figure 7:**
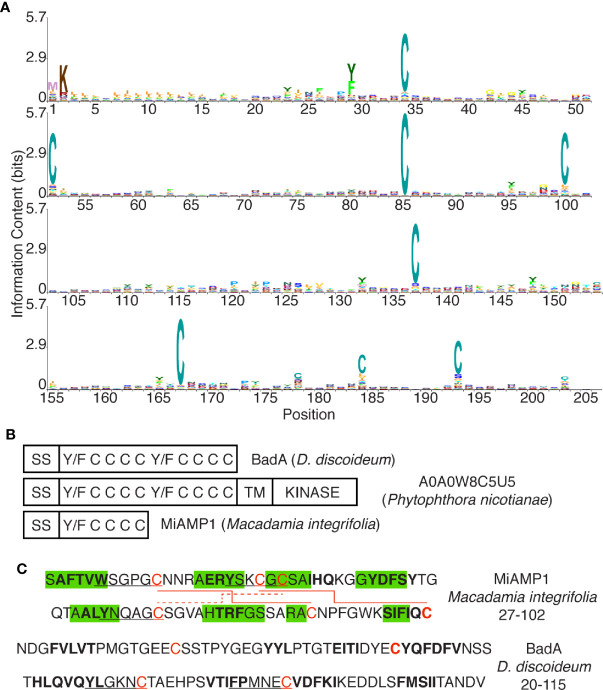
The DUF3430 domain. **(A)** Positions 1 to 205 of the DUF3430 profile HMM from Pfam (PF11912, Pfam 33.1), produced using Skylign (Wheeler et al., 2014) and showing the conserved residues at specific positions. The profile HMM was constructed using *hmmbuild* (default parameters) from HMMER 3.1 on the Pfam full alignment (163 sequences). **(B)** Schematic organization of domains with conserved aromatics and cysteine residues in BadA, a member of a large family of Phytophthora proteins and the plant MiAMP1 protein. **(C)** Sequence and structure of the MiAMP1 protein and the first half of the DUF3430 motif of BadA. Residues shown to be engaged in β-strands are highlighted in green, residues predicted by SOPMA to be engaged in β-strands are in bold, Y/W/FxxxxC motifs are underlined, disulfide bonds are indicated with red lines.

Four fractions eluted from the anion exchange column (150–300 mM NaCl) contained the majority of the lytic activity. They were pooled and further fractionated on a Sephadex size separation column. The activity migrated as a peak with an apparent size between 70 and 30 kDa (**Figure 6A**). We analyzed by mass spectrometry the protein content of three of these fractions, one where the lytic activity was highest (15 ml elution volume), and two where it was significantly lower (13.5 and 16.5 ml). We identified a total of 37 proteins (**Figure 6B**, **Table S1**), including 16 contaminant non-*D. discoideum* proteins. Of the 21 purified *D. discoideum* proteins, five did not exhibit a signal sequence and were not considered further. Of the 16 remaining proteins, 15 were among the 97 identified previously. Eight of them were known lytic enzymes and eight had no known function. Of note, five of the proteins with no known functions (BadA to BadE) belonged to the same family of proteins, characterized by a DUF 3430 domain of unknown function. In order to tentatively identify the protein(s) responsible for the bacteriolytic activity, we applied two successive selections: candidate proteins should be enriched during the first as well as the second purification steps in the fraction where the maximal bacteriolytic activity was observed compared to the fractions with lower levels of activity. This analysis relies on the semi-quantitative information obtained during proteomic analysis, based on the number of peptides of each protein identified in each fraction. Only four proteins met these two criterions: BadA, B and C, as well as phospholipase D (**Table 1**). We tentatively named proteins with the DUF3430 domain Bad, an abbreviation for Bacteriolytic *D. discoideum*. The structure of Bad proteins is presented in **Figure 7** and commented in the Discussion below.

**Table 1:** List of the 16 proteins identified by mass spectrometry.

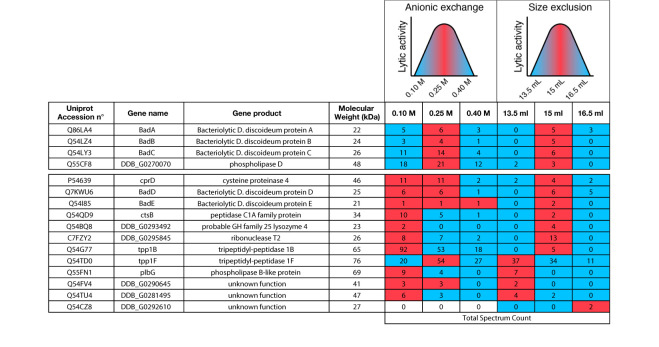

The 16 remaining proteins identified by mass spectrometry after two subsequent rounds of purification are depicted in this table. For each protein, the Uniprot number, as well as its gene name, gene product and predicted molecular weight are indicated. “Total spectrum count” values correspond to the number of peptides of a given protein detected by mass spectrometry in a fraction. In order to identify the protein(s) responsible for the bacteriolytic activity, two successive selections were applied: candidate proteins should be enriched during the first as well as the second purification steps in the fraction where the maximal bacteriolytic activity was observed. This analysis relies on the semi-quantitative information obtained during proteomic analysis, based on the number of peptides of each protein identified in each fraction. The fraction for which each protein is found most abundantly is marked by a red box. Only four proteins met these two criterions: BadA, B and C, as well as phospholipase D.

### Bacteriolytic Activity of BadA *In Vitro* and *In Vivo*

**Figure 8:**
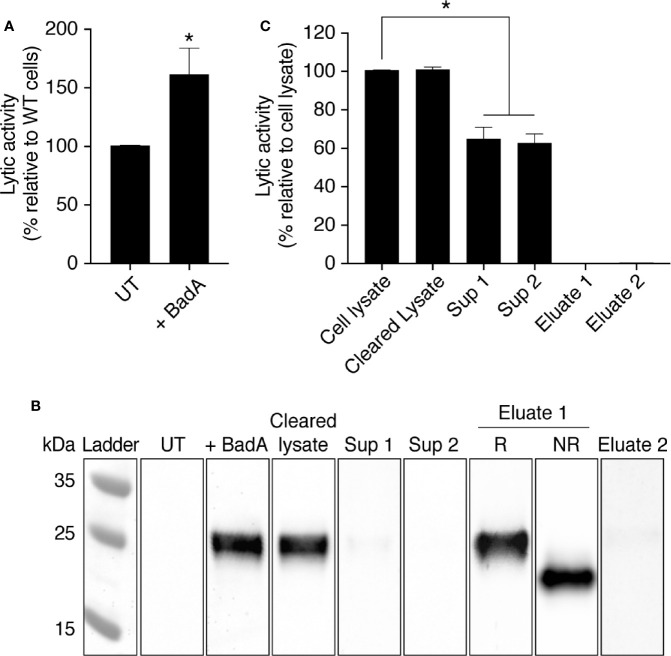
The protein BadA is involved in the lysis of *K. pneumoniae* KpGe bacteria. **(A)** Total cell lysate from untransfected (UT-cells) and BadA-ALFA transfected *D. discoideum* cell were mixed with *Kp* Ge bacteria at pH2 and the bacteriolytic activity was monitored overtime by spectrophotometry at 450 nm. Results are expressed as a percentage of OD_450_ values obtained with untransfected cell lysate after 2 h of incubation (mean ± SEM; N = 10 independent experiments). Cell lysate from BadA-ALFA transfected *D. discoideum* cell was incubated with uncoupled protein A agarose beads. The resulting supernatant (cleared lysate) was subjected to 2 successive immunoprecipitations with the ALFA selector PE resin (Götzke et al., 2019) to obtain respectively supernatants (sup) 1 and 2. Proteins attached to the resin were finally eluted by competition using the ALFA peptide. **(B)** UT and BadA-ALFA transfected cell lysates, as well as the cleared lysate, sup 1, sup 2 and eluates 1 and 2 fractions from IP were separated on an SDS-polyacrylamide gel in reducing (R) or non-reducing (NR) conditions, transferred to a nitrocellulose membrane and revealed using an anti-ALFA antibody. **(C)** BadA-ALFA transfected cell lysate, as well as the cleared lysate, sup 1, sup 2, eluates 1 and 2 fractions from IP were tested for their bacteriolytic activity against *K. pneumoniae* KpGe bacteria. Results are expressed as a percentage of OD_450_ values obtained with transfected cell lysate after 2 h of incubation (mean ± SEM; N = 5 independent experiments; *p < .01; student t test).

In order to test whether Bad proteins are indeed involved in the bacteriolytic activity observed in *D. discoideum* cellular extracts, we overexpressed an ALFA-tagged (Götzke et al., 2019) version of the BadA protein in WT *D. discoideum* cells. BadA has a theoretical isoelectric point of 4.19 and a molecular weight of 19.3 kDa. BadA overexpression resulted in a slight but statistically significant increase of bacteriolytic activity in cell extracts (**Figure 8A**). The tagged protein was detected by western blot in transfected *D. discoideum* cells with an apparent MW of approximately 25 kDa (Guilhen, 2020) (**Figure 8B**). When the cell extract was migrated in an acrylamide gel in non-reducing conditions, the apparent molecular weight of BadA markedly decreased, strongly suggesting that intramolecular disulfide bonds stabilize the folded structure of the BadA protein (**Figure 8B**, NR sample). The ALFA-tagged BadA did not attach to Agarose-Protein A beads and was still present in a cleared cell lysate (Cleared lysate; **Figure 8B**), as was the bacteriolytic activity (**Figure 8C**). The cleared lysate was incubated two times sequentially with antibody-coated beads, and BadA-ALFA was entirely removed from the supernatant after the second incubation as assessed by western blot (Sup1 and Sup2; **Figure 8B**). Concomitantly, a significant fraction of the bacteriolytic activity (37 ± 5%) (N = 5 independent experiments; *: p <.01; student t test) was lost from the lysate (**Figure 8C**, **Supplementary Figure 2**). Thus, in cells overexpressing BadA-ALFA, 40% of the bacteriolytic activity was dependent on BadA-ALFA. Other Bad proteins in the cellular lysate, or the endogenous untagged BadA, may account for the remaining activity. This result demonstrates that BadA contributes to the bacteriolytic activity of *D. discoideum* extracts.

When the purified BadA-ALFA was eluted from the beads, it did not present a measurable bacteriolytic activity (Eluate 1 and Eluate 2; **Figure 8C**). This indicates that other proteins are required for the bacteriolytic activity of *D. discoideum* extracts.

**Figure 9:**
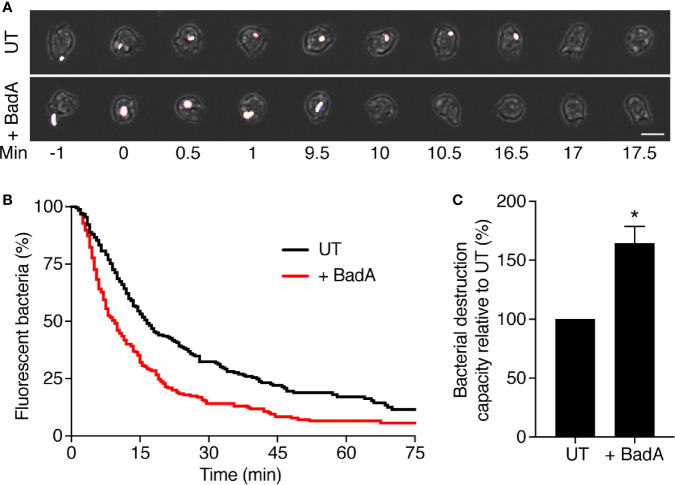
BadA is involved in the lysis of *K. pneumoniae* KpGe bacteria in phagosomes. To visualize ingestion and intracellular destruction of individual bacterial, *D. discoideum* cells were incubated with GFP-expressing *K. pneumoniae* at a ratio of 1:3 for 2 h. Cells were imaged every 30 s by phase contrast and fluorescence microscopy. **(A)** Successive images of untransfected *D. discoideum* cells ingesting (t = 0) and destroying (t = 17 min) individual *K. pneumoniae* bacterium. Below, a BadA-ALFA transfected cell destroyed a *K. pneumoniae* bacterium 10 min after ingestion. Scale bar: 10 μm. **(B)** The time between ingestion and fluorescence extinction was determined for each bacterium and the probability of remaining fluorescent was represented as a function of time after ingestion. Bacterial destruction was analyzed in UT and BadA-ALFA transfected cells. The curves shown were obtained by pooling the results of eight independent experiments. **(C)** Quantification of bacterial destruction capacity of BadA-ALFA transfected cells relative to UT cells. In each independent experiment, a UT control was included, and the bacterial destruction capacity of BadA-ALFA cells was determined using the following formula: (AUC_UT_/AUC_+BadA_)x100; (mean ± SEM; N = 8 independent experiments; total number of events observed: UT = 240, + BadA = 238; *p < .005; student t test).

Finally, in order to verify that BadA can contribute to the destruction of bacteria in living cells, we compared the destruction of ingested *K. pneumoniae* in the phagosomes of WT cells and of WT cells overexpressing BadA-ALFA. As previously described (Leiba et al., 2017), we allowed *D. discoideum* cells to ingest GFP-expressing *K. pneumoniae*, and recorded for each ingested bacteria the time of its ingestion and the time at which its fluorescence was lost (**Figure 9A**). In these experiments we wished to determine if intracellular killing was faster in cells overexpressing BadA than in WT cells. In previous experiments, WT cells grown at a density of 700,000 cells/ml killed ingested *K. pneumoniae* within 3–5 min (Leiba et al., 2017), and it would be exceedingly difficult to determine if killing is accelerated in cells overexpressing BadA. Consequently, we made use of the empirical observation that killing is significantly slower when *D. discoideum* cells are grown at higher densities. In the conditions used in this study (density between 2 and 5.10^6^ cells/ml), the motility and ability to phagocytose were not significantly decreased (**Figure 9A**), while the intracellular killing of ingested *K. pneumoniae* was considerably slowed down: the half-life of ingested bacteria was approximately 17 min (**Figure 9B**). By direct observation, the morphology, motility and phagocytosis of cells overexpressing BadA did not appear grossly altered compared to WT cells (**Figure 9A**). Phagocytosis and macropinocytosis rates were quantified and were indistinguishable in WT and BadA overexpressing cells [92.4 ± 4.5 and 93.4 ± 7.2% of WT (n = 6), respectively]. However, killing of ingested *K. pneumoniae* was faster in cells overexpressing BadA than in untransfected cells (**Figure 9B**). This experiment was repeated 8 times and the difference was reproducible and statistically significant (*: p <.005; student t test) (**Figure 9C**).

## Discussion

The general aim of this study was to identify new *D. discoideum* proteins that may participate in bacterial destruction in phagosomes. We first identified and characterized in *D. discoideum* extracts a bacteriolytic activity against *K. pneumoniae* bacteria. This bacteriolytic activity presumably mimics the destruction of ingested bacteria in phagosomes because: 1- the bacteriolytic activity was only observed at very acidic pH (pH approximately 2), a condition mimicking the very acidic conditions found in *D. discoideum* endosomes and phagosomes (Marchetti et al., 2009; Bodinier et al., 2020); 2- *D. discoideum* extracts lysed more efficiently *K. pneumoniae waaQ* mutant bacteria than the parental bacterial strain. It has previously been shown that *K. pneumoniae waaQ* mutant bacteria are more efficiently killed in *D. discoideum* phagosomes than the parental strain (Lelong et al., 2011); 3- the bacteriolytic activity was markedly lower in *kil1* KO than in WT *D. discoideum* cells. *Kil1* KO *D. discoideum* cells were previously shown to destroy very inefficiently ingested *K. pneumoniae* (Benghezal et al., 2006). In addition, when we purified the bacteriolytic activity, it was found in a cellular fraction highly enriched in lysosomal proteins that would be expected to be present in maturing phagosomes. Four candidate proteins were finally identified that may be involved in the bacteriolytic activity, and three of them (BadA, B, and C) are members of a large but not studied family of dictyostelid proteins, characterized by the presence of a signal sequence for co-translational insertion in the endoplasmic reticulum as well as a conserved domain of unknown function (DUF3430) (El-Gebali et al., 2019).

The DUF3430 (PF11912) domain is found in 160 proteins belonging to the closely related orders of dictyosteliales and acytosteliales, both members of the group of dictyostelids. Genes encoding proteins with a DUF3430 domain are particularly abundant in the *D. discoideum* genome where 54 members of the family are found. In addition, one protein with a DUF3430 domain (Uniprot A0A1Q9F5F2) can be identified in *Symbiodinium microadriaticum* (a dinoflagellate unicellular alga) and one (Uniprot A0A0S4KJV0) in *Bodo saltans* (a flagellated protozoa), although they were not included in the original DUF3430 family of proteins. All DUF3430 domains are preceded by a signal peptide that should target the corresponding protein to the secretory pathway. They exhibit at least eight conserved cysteine residues, and are rich in aromatic residues, and notably contain usually several Y/FxxxxC motifs (**Figure 7A**). In an attempt to identify similar proteins in other species we searched for proteins harboring conserved aromatic and cysteine residues with a spacing similar to that found in the DUF3430 motif. A large family of proteins of unknown function in the *Phytophthora* genus also presents a signal sequence followed by a conserved domain with conserved aromatic and cysteine residues (e.g., Uniprot V9E022), often associated with a transmembrane domain and a kinase cytosolic domain (e.g., A0A0W8C5U5) (**Figure 7B**). Interestingly, a similar sequence is also found in MiAMP1 (Uniprot P80915) from *Macadamia integrifolia*, the founding member of a large family of atypical plant antibacterial peptides (Manners, 2009) (**Figure 7B**). MiAMP1 contains eight β-strands arranged in two Greek key motifs forming a Greek key β-barrel stabilized by disulfide bonds (McManus et al., 1999) (**Figure 7C**) and is thus a member of the larger family of proteins with βγ-crystallin precursor structure (Mishra et al., 2014). When analyzed with SOPMA (Geourjon and Deléage, 1995), the two-dimensional structure of BadA is also predicted to form eight β-strands (**Figure 7C**) and may thus fold into a tridimensional structure similar to that of MiAMP1. Although the significance of these sequence similarities remains to be established, we speculate that Bad proteins may form a tridimensional structure similar to that of MiAMP1 and may act, like MiAMP1 proteins, by permeabilizing bacterial membranes, thus accounting for the bacteriolytic activity of BadA.

Remarkably, the purified BadA entirely failed to cause lysis of *K. pneumoniae* bacteria. This strongly suggests that other factors present in cellular extracts are also required to achieve bacterial lysis. We speculate that, while BadA permeabilizes cell membranes, other phagosomal proteins may be necessary to weaken the protective LPS layers surrounding gram-negative bacteria, or to digest components of the bacterial cell wall. In the future we will try to identify these other factors to gain a more complete understanding of the mechanisms allowing *D. discoideum* proteins to lyse *K. pneumoniae* bacteria.

## Data Availability Statement

The datasets presented in this study can be found in online repositories. The names of the repository/repositories and accession number(s) can be found in the article/**Supplementary Material**.

## Author Contributions

CG and PC conceived and designed the study. CG, WL, EI, XC-Y, and OL performed and analyzed the experiments. CG and PC wrote the manuscript. All authors contributed to the article and approved the submitted version.

## Conflict of Interest

The authors declare that the research was conducted in the absence of any commercial or financial relationships that could be construed as a potential conflict of interest.

## Supplementary Material

Supplementary Figure 1 Protease inhibitors have no effect on the bacteriolytic activity of *D. discoideum* cell lysate. *A*, amoeba cells were lysed with lysis buffer containing either leupeptin (20 μg/ml), aprotinin (10 μg/ml), phenylmethylsulfonyl fluoride (PMSF) (18 μg/ml), iodoacetamide (IAA) (1.8 mg/ml), a mix of the four protease inhibitors or no inhibitors. These lysates [or the lysis buffer alone (no inhib)] were mixed with *K. pneumoniae* KpGe bacteria at pH2 and the bacteriolytic activity was monitored overtime by spectrophotometry at 450 nm. *B*, amoeba cells were lysed with lysis buffer containing or not a commercial protease inhibitor tablet (Pierce #A32963). These lysates [or the lysis buffer alone (no inhib)] were mixed with *K. pneumoniae* KpGe bacteria at pH2 and the bacteriolytic activity was monitored overtime by spectrophotometry at 450 nm. Results are from a single experiment and are expressed as a percentage of OD_450_ at time 0.

Supplementary Figure 2 Immunoprecipitations with untransfected *D. discoideum* cell lysate. Cell lysate from untransfected *D. discoideum* cell was incubated with uncoupled protein A agarose beads. The resulting supernatant (cleared lysate) was subjected to two successive immunoprecipitations with the ALFA selector PE resin to obtain respectively supernatants (sup) 1 and 2. Proteins attached to the resin were finally eluted by competition using the ALFA peptide. Untransfected cell lysate, as well as the cleared lysate, sup 1, sup 2, eluates 1 and 2 fractions from IP were tested for their bacteriolytic activity against *K. pneumoniae* KpGe bacteria. Results are expressed as a percentage of OD_450_ values obtained with untransfected cell lysate after 2 h of incubation. No significant differences were observed between the first 4 conditions (mean ± SEM; N = 7 independent experiments; student t test).
