# Supplementary figures and images for: A New Family of Bacteriolytic Proteins in Dictyostelium discoideum
Source: Front Cell Infect Microbiol. 2021 Feb 3;10:617310. doi: 10.3389/fcimb.2020.617310 (PMC7886984; doi:10.3389/fcimb.2020.617310)

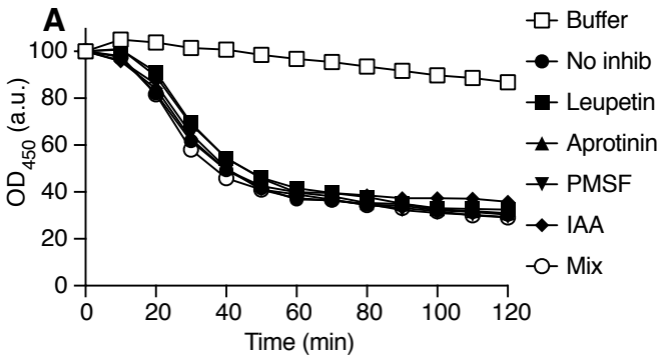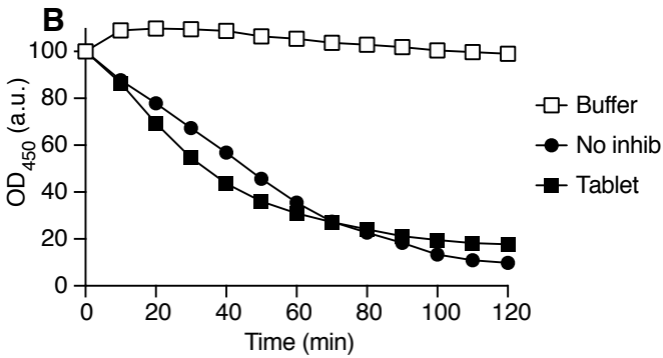

Supplement: Supplementary Figure 1 — Protease inhibitors have no effect on the bacteriolytic activity of D. discoideum cell lysate. A, amoeba cells were lysed with lysis buffer containing either leupeptin (20 μg/ml), aprotinin (10 μg/ml), phenylmethylsulfonyl fluoride (PMSF) (18 μg/ml), iodoacetamide (IAA) (1.8 mg/ml), a mix of the four protease inhibitors or no inhibitors. These lysates [or the lysis buffer alone (no inhib)] were mixed with K. pneumoniae KpGe bacteria at pH2 and the bacteriolytic activity was monitored overtime by spectrophotometry at 450 nm. B, amoeba cells were lysed with lysis buffer containing or not a commercial protease inhibitor tablet (Pierce #A32963). These lysates [or the lysis buffer alone (no inhib)] were mixed with K. pneumoniae KpGe bacteria at pH2 and the bacteriolytic activity was monitored overtime by spectrophotometry at 450 nm. Results are from a single experiment and are expressed as a percentage of OD450 at time 0. [file DataSheet_1.pdf]

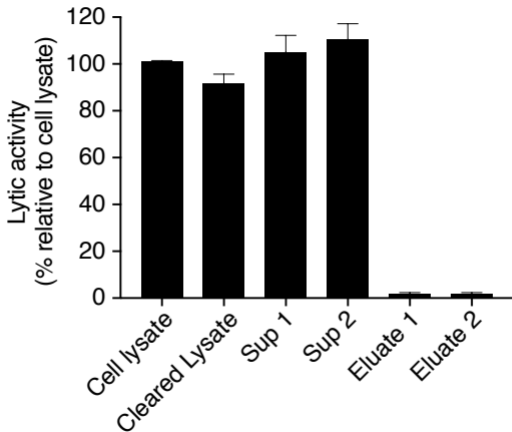

Supplement: Supplementary Figure 2 — Immunoprecipitations with untransfected D. discoideum cell lysate. Cell lysate from untransfected D. discoideum cell was incubated with uncoupled protein A agarose beads. The resulting supernatant (cleared lysate) was subjected to two successive immunoprecipitations with the ALFA selector PE resin to obtain respectively supernatants (sup) 1 and 2. Proteins attached to the resin were finally eluted by competition using the ALFA peptide. Untransfected cell lysate, as well as the cleared lysate, sup 1, sup 2, eluates 1 and 2 fractions from IP were tested for their bacteriolytic activity against K. pneumoniae KpGe bacteria. Results are expressed as a percentage of OD450 values obtained with untransfected cell lysate after 2 h of incubation. No significant differences were observed between the first 4 conditions (mean ± SEM; N = 7 independent experiments; student t test). [file DataSheet_2.pdf]
